# Supplementary material for: Safety of Rigid Bronchoscopy for Therapeutic Intervention at the Intensive Care Unit Bedside
Source: Medicina (Kaunas). 2022 Nov 30;58(12):1762. doi: 10.3390/medicina58121762 (PMC9782846; doi:10.3390/medicina58121762)
Supplement: Supplementary file 1 [file medicina-58-01762-s001.zip › medicina-2039517-supplementary.pdf]

Online supplement

Table S1. Comparison of patient characteristics according to severity of post-procedural complications.

Variables	Severe complication (n = 26)	Non-severe complication (n = 310)	*P*
Location			0.052
Operating room	8 (30.8)	157 (50.6)	
Intensive care unit	18 (69.2)	153 (49.4)	
Age, years	62 (34–77)	63 (50–74)	0.984
Male	11 (42.3)	147 (47.4)	0.616
Body mass index, kg/m^2	20.0 (18.3–24.0)	21.2 (18.6–24.1)	0.628
Current or past smoker	9 (34.6)	129 (41.6)	0.486
Comorbidities			
Cancer	13 (50.0)	128 (41.3)	0.387
Diabetes mellitus	8 (30.8)	89 (28.7)	0.824
Cerebrovascular disease	5 (19.2)	52 (16.8)	0.785
Congestive heart failure	3 (11.5)	50 (16.1)	0.780
Chronic pulmonary disease	1 (3.8)	27 (8.7)	0.710
Chronic liver disease	2 (7.7)	16 (5.2)	0.640
Performance status			0.387
ASA III	1 (3.8)	41 (13.2)	
ASA IV	11 (42.3)	108 (34.8)	
ASA V	14 (53.8)	161 (51.9)	
Tracheostomy before intervention	5 (19.2)	45 (14.5)	0.564
Invasive respiratory support before intervention	13 (50.0)	157 (50.6)	0.950
Mechanical ventilation without ECMO	11 (42.3)	144 (46.5)	0.684
With ECMO	2 (7.7)	13 (4.2)	0.326
Arterial blood gas analysis [a]			
PaO_2 /FiO_2 ratio, mmHg	292 (194–450)	334 (245–445)	0.478
< 200 and/or ECMO	8 (30.8)	58 (18.7)	
200–299	6 (23.1)	72 (23.2)	0.305[b]
≥ 300 and/or no results	12 (46.2)	180 (58.1)	
$PaCO_2$, mmHg	36.8 (29.8–43.4)	38.9 (33.3–43.9)	0.219
HCO_3, mEq/L	23.9 (20.3–28.4)	25.1 (22.3–28.1)	0.232
Reason for intervention			0.898
PITS	12 (46.2)	120 (38.7)	0.455
MCAO	9 (34.6)	107 (34.5)	0.992
POTS	1 (3.8)	23 (7.4)	1.000
Airway FB	1 (3.8)	19 (6.1)	1.000
Relapsing polychondritis	2 (7.7)	15 (4.8)	0.631
PTBS	0	14 (4.5)	0.612
Others[c]	1 (3.8)	12 (3.9)	1.000
Time interval from ICU admission to intervention, hours	24 (16–43)	23 (14–61)	0.827
Site of lesion			0.843

Single lesion	19 (73.1)	232 (74.8)	
Extended lesion	7 (26.9)	78 (25.2)	
Severity of stenosis[d]			0.923
II	9 (34.6)	100 (33.1)	
III	10 (38.5)	128 (42.4)	
IV	7 (26.9)	74 (24.5)	
Procedure details			
Stent insertion	19 (73.1)	202 (65.2)	0.414
Stent change or reposition	12 (46.2)	63 (20.3)	0.002
Tumor removal	1 (3.8)	31 (10.0)	0.491
Stent removal	4 (15.4)	53 (17.1)	1.000
Bougienation only[e]	0	28 (9.0)	0.148
Tracheostomy	0	25 (8.1)	0.239
Foreign body removal	2 (7.7)	18 (5.8)	0.660
Laser cauterization	0	12 (3.9)	0.610
EBV insertion	1 (3.8)	2 (0.6)	0.215
Reversal agents	7 (26.9)	124 (40.0)	0.270
Intervention duration, minutes	30 (20-41)	16 (12-25)	<0.001
Time interval from the end of intervention to the first extubation, minutes [f]	65 (16-886)	85 (15-345)	0.898
≤ 15 minutes	6 (23.1)	74 (23.9)	
16-60 minutes	6 (23.1)	57 (18.4)	0.816[g]
≥ 61 minutes	14 (53.8)	179 (57.7)	
Lengths of ICU stay after intervention, day	6 (3-13)	4 (3-6)	<0.001
Need for additional intervention	13 (50.0)	48 (15.5)	<0.001
ICU mortality	3 (11.5)	13 (4.2)	0.117

[a] Excluding 15 patients receiving ECMO support before the intervention and 18 patients without results of arterial blood gas. However, when classifying the PaO_2/FiO_2 ratio as a categorical variable, patients with ECMO support were categorized as <200 and patients without results were categorized as ≥300.
[b] This *P*-value is for categorized PaO_2/FiO_2 ratios.
[c] Persistent air leak and/or fistula (n = 7), benign tumors (n = 2), inhalation injury (n = 2), and tracheomalacia due to mucopolysaccharidosis (n = 2).
[d] Myer and Cotton grade. There was no stenosis in eight patients in the non-severe post-procedural complication group (persistent air leak and/or fistula = 7, intolerance to tracheal stent due to bronchospasm and mucostasis = 1). They were classified as Grade II.
[e] Bougienation involves balloon dilatation and/or mechanical bougie using a rigid bronchoscope without stent insertion or tumor removal.
[f] There were nine cases with home ventilators (severe post-procedural complication group = 2, non-severe post-procedural complication group = 7) and seven cases who were transferred to other hospitals with mechanical ventilator immediately after the intervention (all were non-severe post-procedural complication group). These cases were classified as ≥ 61 minutes.
[g] This *P*-value is for the categorized variable.
ASA = American Society of Anesthesiologist, ECMO = extracorporeal membrane oxygenation, PaO_2 = arterial partial pressure of oxygen, FiO_2 = fraction of inspired oxygen, $PaCO_2$ = arterial partial pressure of carbon dioxide, PITS = post-intubation or tracheostomy tracheal stenosis, MCAO = malignant central airway obstruction, POTS = postoperative tracheobronchial stenosis, FB = foreign

body, PTBS = post-tuberculous tracheobronchial stenosis, ICU = intensive care unit, EBV = endobronchial valve.
